# Ocular injuries associated with two-wheeled electric transportation devices and motorcycle accidents

**DOI:** 10.1038/s41598-022-23860-z

**Published:** 2022-11-29

**Authors:** Omer Lev Ari, Gad Shaked, Tal Michael, Adi Givon, Moran Bodas, Israel Trauma Group, A. Acker, N. Aviran, H. Bahouth, A. Bar, A. Becker, A. Braslavsky, D. Fadeev, A. L. Goldstein, I. Grevtsev, I. Jeroukhimov, A. Kedar, A. Korin, B. Levit, A. D. Schwarz, W. Shomar, D. Soffer, I. Schrier, M. Venturero, M. Weiss, O. Yaslowitz, I. Zoarets, Erez Tsumi

**Affiliations:** 1 Faculty of Health Sciences, Joyce and Irving Goldman Medical School, Ben Gurion University of the Negev, Beersheba, Israel; 2 Department of Ophthalmology, Soroka University Medical Center, Ben Gurion University of the Negev, Beersheba, Israel; 3 Department of General Surgery and Trauma Unit, Soroka University Medical Center, Ben-Gurion University, Beersheba, Israel; 4 Department of Epidemiology, Biostatistics, and Community Health Sciences, The Faculty of Health Sciences, Ben-Gurion University of the Negev, Beersheba, Israel; 5 Israel National Center for Trauma and Emergency Medicine Research, The Gertner Institute for Epidemiology and Health Policy Research, Sheba Medical Center, Ramat Gan, Israel; 6 Department of Emergency and Disaster Management, School of Public Health, Faculty of Medicine, Tel-Aviv University, Tel-Aviv-Yafo, Israel; 7 Soroka University Medical Center, Beersheba, Israel; 8 Rambam Medical Center, Haifa, Israel; 9 Kaplan Medical Center, Rehovot, Israel; 10 Haemek Medical Center, Afula, Israel; 11 Ziv Medical Center, Safed, Israel; 12 Barzilai Medical Center, Ashkelon, Israel; 13 Wolfson Medical Center, Holon, Israel; 14 Yoseftal Medical Center, Eilat, Israel; 15 Itzhak Shamir Medical Center, Be’er Ya’akov, Israel; 16 Hadassah Ein Kerem Medical Center, Jerusalem, Israel; 17 Sheba Medical Center, Ramat Gan, Israel; 18 Hillel Yaffe Medical Center, Hadera, Israel; 19 Poriya Medical Center, Tiberias, Israel; 20 Shaare Zedek Medical Center, Jerusalem, Israel; 21 Nazareth Hospital EMMS, Nazareth, Israel; 22 Tel Aviv Sourasky Medical Center, Tel Aviv-Yafo, Israel; 23 Rabin Medical Center, Petah Tikva, Israel; 24 Laniado Medical Center, Netanya, Israel; 25 Western Galilee Medical Center, Nahariya, Israel; 26 Meir Medical Center, Kfar Saba, Israel; 27 Assuta Ashdod Hospital, Ashdod, Israel

**Keywords:** Public health, Medical research, Risk factors

## Abstract

Electric bicycles and scooters have gained popularity among riders; studies assessing these device-related injuries have not specified ocular trauma. Our study examined the types and risk factors for ocular and periocular injuries associated with electric devices compared to motorcycle accidents. The study was conducted on the National Trauma Registry database from 20 trauma centers, including patients involved in accidents with electric bicycles, scooters, and motorcycles between 2014 to 2019. Injured riders were assigned into two groups: motorcycle group (M) and electric bicycle & scooter group (E). Data such as gender, age, protective gear use, ocular injury type, injury severity score (ISS), and ocular surgery were captured. Logistic regression models were conducted for injury types and the need for surgery. 8181 M-riders and 3817 E-riders were involved in an accident and hospitalized. E-riders suffered from ocular injury more than M-riders. Males were most vulnerable and the ages of 15–29. Orbital floor fracture was the most common injury, followed by ocular contusion, eyelid laceration, and other ocular wounds. Electric bicycle and scooter riders are more likely to suffer from ocular injury than motorcycle riders. Riders without helmets are at greater risk for injuries, specifically orbital floor fractures. ISS of 16 + was associated with injury demanding ocular surgery.

## Introduction

Two-wheeled electric transportation devices, such as electric bicycles and scooters, have gained popularity among city riders in the last decade, along with the use of motorcycles, as a means to avoid traffic on crowded roads^1^.

While four-wheeled vehicles and motorcycle safety systems have improved dramatically over the years, two-wheeled electric device riders have been left exposed to traffic hazards with no significant improvement in their safety systems^2^. The unsatisfactory safety systems combined with the increase in travel speed of modern two-wheeled vehicles have led to a substantial increase in the risk of severe injuries among these road users^3^.

Though injuries resulting from motorcycle accidents have been well studied and described in the literature, there is only partial information on injuries resulting from accidents involving two-wheeled electric devices. Recent evidence has indicated that accidents occurring with two-wheeled electric devices primarily cause head and limb injuries^4^.

It is well established that motorcycle riders involved in motor vehicle accidents (MVAs) have a higher risk for fatal injuries due to the motorcycle engine capacity and the kinematics of the accident. As for motorcycle-related injuries, one of the most frequently sustained injuries found in previous studies is head and facial injury^5^. Traffic accidents involving motorcycle riders have been a major external cause of ophthalmologic injury necessitating hospitalization^6^.

Ocular injuries following motorcycle accidents mainly affect the conjunctiva, eyelids, and corneas and present with traumatic hemorrhage or laceration. Those injuries can coexist with head injuries and maxillofacial fractures, such as zygomatic or mandibular bone fractures, predominantly occurring without protective gear use^7^.

Powered scooters and electric bicycle-related injuries include soft tissue injuries and fractures^8^. As for ocular injuries, eyelid injuries are the third most frequently sustained soft tissue injury, and orbital fractures are the most common osseous injuries^9^. Lateral orbital rim and floor fractures have been found to have the highest prevalence, followed by orbital roof and medial wall fractures. While ocular trauma associated with two-wheeled powered device use is not well reported, the possibility of suffering a significant ocular injury while riding those devices has been noted in the literature^10^.

Ocular injuries impose a substantial burden on emergency departments and are associated with prolonged medical implications and a clear deterioration in patients' quality of life. Ocular trauma by itself leads to a vast number of visits to the emergency department every year^11^. Studies assessing injury characteristics related to two-wheeled electric transportation devices have not specified ocular trauma and lacked information on the types of ocular injuries^8,9,12–14^.

The hypothesis of this study is that there are differences in the characteristics of ocular injuries between the two two-wheeled vehicles due to the different engine power, use of protective gear, and traffic habits, taking into account that there may be other factors not already identified. Our study aims to identify the types of ocular injuries associated with two-wheeled electric transportation devices, characterize the risk factors for these injuries, and compare them to those presented among victims of motorcycle accidents.

## Materials and methods

### Study design

This retrospective cohort study was conducted on the Israeli National Trauma Registry (INTR) database from twenty trauma centers in Israel, including patients involved in accidents while riding electric bicycles, scooters, and motorcycles and hospitalized between 2014 to 2019. Injured riders were assigned into two groups: motorcycle group (M), electric bicycle, and scooter group (E). Background characteristics, including gender and age, as well as data regarding the accident; protective gear use, seasonality, type of ocular injury, injury severity score (ISS), and ocular surgery, were captured.

### Israel National Trauma Registry (INTR)

Data were collected at the hospitals by the trauma registrars, monitored by the trauma coordinator, and supervised by the trauma unit director. Data is transmitted via a computerized system to the Gertner Institute's central database, managed by the Israel National Center for Trauma and Emergency Medicine Research. The trauma registry database consists of all injuries classified ICD-9-CM, with diagnosis codes. The registry records all patients hospitalized, including those who died in the Department of Emergency Medicine or were transferred to another hospital following an injury. The information collected on each patient comprises demographic data, injury circumstances (riding two-wheeled transportation device, protection gear use), injury type and severity (ocular injuries, additional injuries), and surgical procedures. To receive the relevant data from Gertner Institute, a formal request has been submitted to Gertner. Upon receiving, Gertner's data does not include the trauma patients' personal information^1,15^.

### Statistical analysis

The Chi-square test was used to calculate proportional differences between the groups that used two-wheeled electric transportation devices and motorcycles. Multivariate logistic regression models assessed the adjusted effect of variables. The overall significance level was set to 0.05. The statistical analysis was carried out using SAS software, version 9.4, SAS Institute, Cary, NC.

### Ethics approval and consent to participate

The research received the approval of the Sheba Medical Center's Institutional Research Ethics Committee (5138–18-SMC). The study is based on an anonymous registry; therefore, the need for informed consent was waived. The research was preformed in accordance with relevant guidelines and regulations following the Declaration of Helsinki.

## Results

A total of 11,998 patients were included in the final analysis: motorcycle riders (*n* = 8181) and two-wheeled electric transportation device riders (*n* = 3817). All were involved in accidents and hospitalized between the years 2014–2019. 282 two-wheeled electric transportation device riders and 310 motorcycle riders sustained ocular and periocular injuries during the study period.

Electric bicycle and scooter riders suffered more from orbital, periorbital, or ocular injury than motorcycle riders (M 3.8%, E 7.4%, *p* < 0.0001). Males were most vulnerable (M 93.2%, E 89.7%, *p* = 0.125). The age distribution of the groups was significantly different (*p* < 0.0001). Most vulnerable were the ages of 15–29 (M 64.2%, E 37.2%), followed by the ages of 30–44 (M 20.3%, E 23.4%,), the ages of 45–59 (M 10.7%, E 12.8%), and 60–74 (M 4.2%, E 9.2%). Unlike group M, 48 riders in group E were under the age of 14 (M 0, E 17%). Only a fraction of the riders in each group were 75 and above (M 0.7%, E 0.4%).

Data for protective gear were available for most riders; 88 motorcycle riders and 107 two-wheeled electric device riders had no helmet use data (M 28.4%, E 37.9%, *p* < 0.0001). Motorcycle riders used more helmets (M 62.9%, E 6%, *p* < 0.0001), Only 27 riders from group M were without protective equipment, compared to 158 in group E riders, in which the majority were without (M 8.7%, E 56%, *p* < 0.0001). The registry does not specify the helmet type. As for seasonality, there was no statistically significant season in which accidents that led to an ocular injury occurred more. Winter was the least common season for ocular injuries accidents (M 19.7%, E 17%, p = 0.219). The detailed demographic data are shown in Table 1.

**Table 1:** Characteristics of the study injured population.

Variable	M group	E group	*P*-value
	*n* = 310	*n* = 282	
**Ocular injury**			< .0001
Injured	310 (3.8%)	282 (7.4%)	
Not injured	7871 (96.2%)	3535 (92.6%)	
**Gender**			0.125
Female	21 (6.8%)	29 (10.3%)	
Male	289 (93.2%)	253 (89.7%)	
**Age**			< .0001
0–14	0	48 (17%)	
15–29	199 (64.2%)	105 (37.2%)	
30–44	63 (20.3%)	66 (23.4%)	
45–59	33 (10.7%)	36 (12.8%)	
60–74	13 (4.2%)	26 (9.2%)	
75 +	2 (0.7%)	1 (0.4%)	
**Protective gear**			< .0001
Helmet	195 (62.9%)	17 (6%)	
Without helmet	27 (8.7%)	158 (56%)	
Unknown	88 (28.4%)	107 (37.9%)	
**Seasonality**			0.219
Summer	77 (24.8%)	82 (29.1%)	
Autumn	79 (25.5%)	84 (29.8%)	
Winter	61 (19.7%)	48 (17%)	
Spring	93 (30%)	68 (24.1%)	

Most of the ocular injuries were combined with additional injuries (M 98.7%, E 95.7%, *p* = 0.026). Orbital floor fracture was the most common injury (M 71.3%, E 61.7%, *p* = 0.013), followed by ocular contusion (M 25.2%, E 29.1%, *p* = 0.284), eyelid laceration (M 13.6%, E 17.7%, *p* = 0.161) and other ocular wounds (M 8.1%, E 6.7%, *p* = 0.539). Group M suffered more from high-severity injuries, ISS 16 and above, than group E (M 63.9%, E 35.8%, *p* < 0.0001). Only a small portion of the riders had suffered an injury requiring ocular surgery (M 5.2%. E 3.6%, *p* = 0.338). The detailed injury characteristics data are shown in Table 2.

**Table 2:** Characteristics of the injury.

Variable	M group	E group	*P*-value
	*n* = 310	*n* = 282	
**Type of injury**			0.026
Eyes only	4 (1.3%)	12 (4.3%)	
Eyes + Other areas	306 (98.7%)	270 (95.7%)	
Type of ocular injury*			
Orbital floor	221 (71.3%)	174 (61.7%)	0.013
Eyelid laceration	42 (13.6%)	50 (17.7%)	0.161
Ocular contusion	78 (25.2%)	82 (29.1%)	0.284
Other ocular wounds	25 (8.1%)	19 (6.7%)	0.539
**ISS**			< .0001
1–14	112 (36.1%)	181 (64.2%)	
16 +	198 (63.9%)	101 (35.8%)	
Need of ocular surgery	16 (5.2%)	10 (3.6%)	0.338

*Each patient may have more than one injury.

Table 3 contains the results of logistic regression for ocular injury. The risk of suffering an ocular injury (orbital, periorbital, or ocular injury) due to an accident while riding two-wheeled transportation in group E was 1.4-fold than group M (OR = 1.4; *p* = 0.004). The risk for ocular injury was 2.4-folds higher among riders who did not use protective gear (OR = 2.4; *p* < 0.0001). No gender was at greater risk for an ocular injury (OR = 1.2; *p* = 0.266). Riders aged 15–29 were more prone to ocular injuries than younger riders (OR = 1.4; *p* = 0.056). No association was found between a specific season of the year and the risk of suffering an ocular injury.

**Table 3:** Multivariate analysis- Logistic Regression for ocular injury.

Variable	OR	95% CI	*P*-value
**Gender**			
Male vs. Female	1.2	0.887–1.621	0.266
**Age**			
15–29 vs 0–14	1.4	1.001–1.934	0.056
30–44 vs 0–14	1.4	0.975–1.995	0.074
45 + vs 0–14	1.4	0.965–1.996	0.083
**Protective gear**			
Without helmet vs. helmet	2.4	1.814–3.157	< .0001
**Seasonality**			
Autumn vs. Summer	1.1	0.839–1.317	0.663
Winter vs. Summer	1	0.779–1.288	0.979
Spring vs. Summer	1.2	0.919–1.445	0.219
**Type of 2-wheeled device**			
Electric bicycle and scooter vs. Motorcycle	1.4	1.116–1.770	0.004

The multivariate analysis for orbital floor fracture is presented in Table 4. Riding with no protective gear was associated with an increased risk of orbital floor fractures in both groups (OR = 3.3; *p* < 0.0001). Riders aged 15–29 were more vulnerable to orbital floor fractures than younger riders (OR = 2; *p* = 0.003). No gender was at greater risk of suffering from an orbital fracture (OR = 1.3; *p* = 0.201). No association was found between a specific season of the year and the risk of suffering an orbital floor fracture. No group was associated with an increased risk of orbital floor fracture (OR = 1.1; *p* = 0.702).

**Table 4:** Multivariate analysis- Logistic Regression for orbital floor fracture.

Variable	OR	95% CI	*P*-value
**Gender**			
Male vs. Female	1.3	0.891–1.918	0.201
**Age**			
15–29 vs 0–14	2.01	1.294–3.271	0.003
30–44 vs 0–14	1.99	1.240–3.334	0.006
45 + vs 0–14	2.02	1.250–3.394	0.006
**Protective gear**			
Without helmet vs. helmet	3.3	2.356–4.582	< .0001
**Seasonality**			
Autumn vs. Summer	1.1	0.838–1.448	0.491
Winter vs. Summer	1	0.733–1.356	0.998
Spring vs. Summer	1.2	0.890–1.545	0.258
**Type of 2-wheeled device**			
Electric bicycle and scooter vs. Motorcycle	1.1	0.800–1.392	0.702

The risk for ocular injury demanding surgery was 5.8 folds higher with an ISS of 16 and above (OR = 5.8; *p* < 0.0001). None of the groups was associated with an increased risk of ocular injury demanding surgery (OR = 0.9; *p* = 0.846). No age group was more likely to suffer an injury requiring ocular surgery (as shown in Table 5). The lack of helmet use was not associated with a need for ocular surgery (OR = 2.5; *p* = 0.309).

**Table 5:** Multivariate analysis- Logistic Regression for the need for ocular surgery.

Variable	OR	95% CI	P-value
**ISS**			
1–14 vs. 16 +	5.8	2.666–12.971	< .0001
**Age**			
15–29 vs 0–14	0.9	0.209- 5.950	0.868
30–44 vs 0–14	1.5	0.349–10.596	0.609
45 + vs 0–14	0.5	0.082–4.254	0.506
**Protective gear**			
Without helmet vs. helmet	2.5	0.678–8.837	0.309
**Type of 2-wheeled device**			
Electric bicycle and scooter vs. Motorcycle	0.9	0.309- 2.554	0.846

## Discussion

Our study aimed to examine the types and risk factors for periocular and ocular injuries associated with two-wheeled electric transportation devices compared to motorcycle accidents, using a large-scale database from 20 trauma centers. Eventually, we hope the study results may lead to recommendations for future actions (i.e., legislative, identifying targeted population, need to introduce innovative protective gear) to reduce these types of injuries.

We found that two-wheeled electric device riders are 1.4-fold more prone to periocular or ocular trauma in the event of an accident than motorcycle riders. Furthermore, even though motorcycle riders suffer from an overall higher injury severity, electric device riders endure more from ocular injuries. Orbital floor fracture was the most common injury, followed by ocular contusion, eyelid laceration, and other ocular wounds. Our findings are consistent with previous reports about craniofacial injuries among electric scooter users^9,10^. One explanation could be the low incidence of helmet wearing in the electric bicycle and scooter; 56% of the injured riders in group E were without helmets. The helmet type might also affect these results; Motorcycle riders tend to use a full-face helmet that provides better ocular surface protection. Two-wheeled electric device riders tend to use less protective helmets, such as open-face or half-coverage helmets^16^.

Another explanation is a higher gravity center for electric scooter riders compared to seated vehicles; electric-device riders cannot stand by themselves and tend to fall when there is a loss of balance. Two-wheeled electric devices users ride at a lower velocity than motorcyclists, impacting the pattern of their injuries; E riders may fall or slip from their device, therefore experiencing milder injuries such as ocular and ocular adnexal injuries, while motorcyclists tend to collide with objects (moving or static), suffering more diverse injuries in multiple body sites. Moreover, balance loss and slipping mechanisms of injury make the prominent facial bones more susceptible to injury. The sliding mechanism, characterizing electric device rider's accidents, can result in falling on one side, increasing the vulnerability of the orbital bones and subjecting the orbit adnexa to an injury^17^.

Riders aged 15–29 comprised the age group that sustained the largest proportion of ocular injuries. One possible explanation is their inclination for dangerous driving habits and lack of experience in road driving compared to other age groups^12,18^. Children comprising the injured riders in the age group of 0–14, were solely electric device riders; consisting 17% of the riders who sustained periocular or ocular trauma in group E. This age group is more vulnerable to ocular trauma due to their curiosity, immature motor abilities, juvenile judgment skills, and the habit of imitating with no concerns for dangers and consequences. Visual development terminates at the age of 10; therefore, ocular trauma at a young age has a poor prognosis despite adequate therapeutic measures compared to adults. Ocular and periocular injuries in children may lead to visual impairment due to increased ocular inflammatory response, inadequate treatment compliance, and propensity to develop amblyopia. Visual impairments affect emotional and mental development, causing psychological, occupational, and financial difficulties to the child and his family^19^. Unintentional injuries amongst children and adolescents, such as injuries following accidents, can lead to developmental years lost; soft tissue injuries in this age group might result in delayed psycho-social consequences extending beyond the injury itself^20^.

The fact that most of the ocular and periocular injured riders were young with no protective gear should be highly alarming, indicating that the importance of helmet wearing is not rooted amongst the younger generation, necessitating immediate attention in education, awareness-raising, and improvement in law enforcement. Our study demonstrated a higher incidence of protective gear usage among motorcycle riders at the time of the accident than electric bicycle and scooter riders. This can be clarified by more vigilant law enforcement upon motorcyclists and better awareness of motorcycle riders' danger of not using helmets^21^. The low percentage of helmet use in the electric group might suggest that these transportation devices are perceived as harmless. Similar helmet use rates among electric scooter riders were documented in other reports^10,13^. The risk for ocular injury is significantly higher when no protective gear is used in the accident. These findings are compatible with previous literature examining helmet use and the risk of suffering a head injury^14^.

Moreover, riding a motorcycle in Israel requires a driving license, necessitating formal driving education, a theoretical exam, and a practical driving test. Up until 2019 (the last year of our study), riding an electric device did not require any of the above. From 2019 forward, upon changing legislation in the matter, a mandatory written exam is warranted to ride these devices. The minimum age for driving a motorcycle is 16; in practice, most people do not obtain a driving license before 17.5 years. As mentioned above, while motorcyclists drive under more extensive law enforcement, E riders have little to non, granting them freedom of law obedience to their judgment^22^. Negligent driving behavior, lack of safety knowledge, a more aggressive driving nature, and rule-breaking tendency expose E-riders to traumatic injuries in accidents^23^. While the law is clear for motorcycle mandatory helmet use and strictly enforced, it was only recently enacted for two-wheeled electric devices, and enforcement is still lacking^24–27^.

ISS is an anatomy-based scoring system that computes the global score for patients with numerous injuries to measure and classify trauma severity. We demonstrated a correlation between trauma severity, i.e., a high ISS score, and the likelihood of ocular trauma; a similar correlation was shown by Goyal et al.^28^. The device's velocity differences impact the injury site; a higher driving velocity exposes riders to numerous bodily injuries. Our findings confirm that most injured riders who suffered from ocular injuries had additional damage in other body areas.

In our study, only a small number of riders had suffered an injury requiring ocular surgery. This is a lower incidence of surgical interventions than previous studies examining other types of injuries following two-wheeled accidents. It might be due to the mixed site's pattern of injuries. Surgical interventions for fractures of the zygomatic bone, including the orbit area, might be treated in different hospitals at different departments, such as the Oral and Maxillofacial surgery department, and therefore not included as an ophthalmic surgery in the data collected for this study^17^.

Similar to former studies regarding protective gear and facial injuries^12^, we established an association between riding with no protective gear and orbital floor fractures. Orbital fractures were previously associated with optic nerve injuries in patients with significant trauma and with visual impairments. Vision losses might delay recovery and have significant occupational and financial effects on an individual^29^. Riders aged 15–29 were more vulnerable to orbital floor fractures than younger riders; this corresponds with the common notion discussed earlier.

As for the risk of injury necessitating ocular surgery, riders with an ISS of 16 and above were more likely to require ocular surgery. To the best of our knowledge, this is the first time ISS and ocular surgery were correlated. A correlation between ISS and the need for surgery, in general, has been described before^30^.

Protective gear usage and the type of transportation device were not associated with the need for ocular surgery; it can be clarified by the correlation found between a high ISS and the need for ocular surgery. The helmet and type of two-wheeled transportation device variables contribute to a higher ISS outcome, resulting in their significance loss in the multivariate regression.

Our data does not specify other parameters, such as other vehicles involved or road-related factors, which could assist in defining future strategies. Additional studies are required to assess their contribution.

Traumatic ocular injuries, such as those caused while riding two-wheeled devices, burden emergency departments, resulting in prolonged medical consequences and decreased quality of life^11^. Reducing the number of these accidents through educational programs, better law enforcement, and changes in legislation might assist in diminishing its burden on the national health system. Authorities should encourage the development of innovative protective gear to minimize these accidents' outcomes and healthcare costs.

## Conclusions

Electric bicycle and scooter riders are more likely to suffer from ocular injury than motorcycle riders, possibly due to the underuse of helmets in this group. Riding a two-wheeled electric transportation device was associated with an increased risk for orbital, periorbital, or ocular injury. Riders without protective gear were at greater risk for ocular and periocular injuries, specifically orbital floor fractures. Riders of the age group of 15–29 years are more prone to this type of accident. As motorcycle, electric bicycle, and scooter accidents rise, our results regarding ocular injuries suggest the need for authorities' engagement in preventing these accidents.

## Limitations

The data used for this study were collected and submitted by various hospital trauma registers. Once data is obtained from the hospital and entered into the central database, logical and other assessments are made to guarantee quality. Missing, unclear, or incorrect information is corrected and completed. The data does not include records relevant to the study, such as patients who passed away in the incident area or on the way to the hospitals. Furthermore, the data only includes cases of patients admitted to the E.R. and hospitalized directly after the incident and in the 72 h following it, meaning that cases relevant to this study that received medical attention 72 h or more after the incident were not included. The data does not specify the setting in which the accident occurred. Whether the rider abused drugs or alcohol, and what was the mechanism of the accident. The records do not specify the rider's protective gear other than the helmet; some helmets are better at preventing facial injuries^16^. Due to the study's retrospective design, it might be essential to perform a prospective study and gather the missing data to derive conclusions regarding the missing information.

## Data Availability

The datasets generated and analyzed during the current study are not publicly available due to hospitalization privacy but are available from the corresponding author upon reasonable request.
